# Rationale and protocol for a systematic review and meta-analysis on reduced data gathering in people with delusions

**DOI:** 10.1186/2046-4053-3-44

**Published:** 2014-05-08

**Authors:** Peter Taylor, Paul Hutton, Robert Dudley

**Affiliations:** 1 Institute of Psychology, Health and Society, University of Liverpool, Liverpool L69 3BX, UK; 2 School of Health in Social Care, University of Edinburgh, Old College, South Bridge, Edinburgh EH8 9YL, UK; 3 Doctorate of Clinical Psychology, Newcastle University, Newcastle upon Tyne NE1 7RU, UK; 4 Early Intervention in Psychosis service, Northumberland Tyne and Wear NHS Foundation Trust, Northumberland, UK

**Keywords:** Jumping to conclusions, Decision-making, Psychosis, Delusions, Beads task, Meta-analysis, Protocol

## Abstract

**Background:**

The tendency to form conclusions based on limited evidence is known as the ‘jumping to conclusions’ (JTC) bias, and has been a much studied phenomena in individuals with psychosis. Previous reviews have supported the hypothesis that a JTC bias is particularly linked to the formation and maintenance of delusions. A new systematic review is required as a number of studies have since been published, and older reviews are limited by not systematically assessing methodological quality or the role of study design in influencing effect size estimates. This review aimed to investigate if there is an association between psychosis or delusions and JTC bias.

**Methods:**

The current protocol outlines the background and methodology for this systematic review and meta-analysis. Eligible articles will be identified through searches of the electronic databases PsycInfo, PubMed and Medline using relevant search terms, supplemented by hand-searches of references within eligible articles and key review articles within the field. Eligibility criteria were as follows: studies must recruit individuals with: i) schizophrenia spectrum conditions or ii) experiences of delusions. Case-control, cross-sectional, observational and prospective designs will be included but treatment trials and experimental studies excluded. Studies must use the beads task to assess JTC or a conceptually equivalent task. The outcomes will be the average number of ‘draws to a decision’ in the beads task (or related variant) and the proportion of the sample judged to demonstrate a JTC bias. Literature searches, study selection, data extraction, risk of bias assessment and outcome quality assessment will be undertaken by two independent reviewers. Meta-analyses will be undertaken for continuous (mean number of ‘draws to a decision’) and binary outcomes (number of people classified as having JTC bias).

**Discussion:**

Understanding of the size of the JTC effect and the contexts within which it occurs is important both in terms of informing models of delusional thinking and in guiding treatments for those with delusions or psychosis. However, a definitive, up-to-date review and meta-analysis of the JTC bias is currently lacking. The proposed review will fill this gap and resolve key issues regarding the factors which moderate the JTC bias.

**PROSPERO registration:**

CRD42014007603 http://www.crd.york.ac.uk/PROSPERO/display_record.asp?ID=CRD42014007603

## Background

There is evidence that people with delusional beliefs, usually in the context of a psychotic illness, make decisions on the basis of less evidence than people without such beliefs [1]. Consequently, people with delusions have been described as having a ‘jumping to conclusions’ (JTC) bias [2]. Such reasoning biases may contribute to the formation of a delusional belief in so far as the belief is formed on the basis of little evidence, without considering alternatives, or looking for further information [3].

This JTC finding has been demonstrated repeatedly, usually on a measure of probabilistic reasoning called the beads task. In this task, participants are presented with two jars containing coloured beads in equal but opposite ratios. For example, the jars may contain 85 black beads and 15 red and *vice versa*. Participants are told that one of the jars has been chosen and beads from the jar will be presented one at a time, and that their task is to decide when they know with certainty whether it is the mainly black bead jar or the mainly red bead jar that has been selected.

A number of studies have suggested that, as a group, people with delusions require fewer beads before making a decision, or fewer ‘draws to a decision’ (DTD), than clinical and non-clinical controls [2,4]. A second outcome arising from the beads task is extreme responding (or JTC), which has been defined as deciding on the basis of two or fewer beads [5]. According to this criterion, around 40 to 70% of people with delusions seem to demonstrate JTC, compared to around 10 to 20% of clinical and non-clinical participants [6]. Similar biases have been shown on other tasks that also involve the gathering of information required for a decision [7,8].

The beads task typically uses material unrelated to the content of the delusional belief. This is valuable as if people were to reason about their delusional beliefs it could lead to tautological explanations. However, clearly delusions tend to have a limited number of themes, and in the case of grandiose and persecutory delusional beliefs which are particularly common, the theme is around whether a person is liked or valued, and is essentially about their worth [9]. Consequently, some researchers have manipulated the content of the beads task to reflect more emotionally salient themes (for example, indicating that the task is to decide if a ‘person very much like yourself is liked by other people’). Using such methods there has been some indication that it may increase the data gathering bias and/or lead to more errors in decision- making [1].

The suggestion that delusions may involve a hasty data gathering style, which precludes consideration of alternative explanations, has been incorporated in a number of multifactorial models of delusion formation and maintenance; for example, see [9]. Consequently, this data gathering style is specifically targeted in a number of emerging treatment packages in order to help people overcome this style and possibly to improve the efficacy of Cognitive Behavioural Therapy (CBT) for delusional beliefs [10].

In the only existing meta-analysis, Fine and colleagues [4] concluded that the best measure of the data gathering bias is DTD. They also noted that a tendency to gather less evidence in the beads task is reliably associated with the presence of delusional symptomatology, but that emotionally salient content had no discernible effect on data gathering. This was a helpful review but was limited in a number of ways. First, there was a limited literature available at the time. There were 12 clinical studies, and two non-clinical studies (using participants who scored high or low for delusional ideation). Similarly, in relation to the impact of content on decision-making there were only three studies using salient materials. Second, at that time the studies were characterised by small sample sizes and, third, there was no consideration of methodological quality. This is problematic as guidelines for reporting in meta-analyses emphasise the importance of methodological quality assessment [11] as this can have a substantial impact on the interpretation of results and the conclusions drawn. Fourth, the meta-analysis violated the assumptions of statistical independence by combining non-independent effects, which is advised against due to the impact on the standard error of the aggregated effect [12].

Since that review, there has been a proliferation of studies with some 40 or so being reported using clinical samples and some 12 or more using non-clinical participants [3]. The sample sizes in these studies have increased [13]. In addition, the range of groups that have been considered has expanded so that studies have been undertaken with people in their first psychotic episode [14], as well people in more acute, chronic and remitting states [15,16].

A recent review updated the evidence but did not deploy meta-analysis or investigate whether methodological quality moderated the findings [3]. Hence it would seem timely to revisit the literature. The goal of the proposed review and meta-analysis will be to provide a robust and up-to-date account of the true size of the JTC effect in relation to delusions and psychosis, considering a variety of theoretically informed moderators and providing an assessment of the effect of study quality on overall estimates.

### Aims and scope of this review

The main aim of this review is to consider the extent to which people with non-affective psychosis, and specifically those with delusions, demonstrate reduced data gathering relative to psychiatric and non-clinical controls. We will investigate (a) whether there are a fewer number of DTD for individuals diagnosed with a psychotic disorder versus individuals without these experiences, (b) whether significantly more people with non-affective psychosis demonstrate a JTC style relative to control participants; (c) a key question is whether the bias is specific to delusions, or whether it is more general to psychosis, and we will therefore also test whether DTD and JTC style differ between those experiencing delusions versus people who have psychosis but do not report delusions.

Two broad sets of secondary questions will address a) the impact of illness variables and b) the impact of task variables on data gathering in people with non-affective psychosis. Since the focus of this review is on people with clinical levels of psychosis, we will not examine differences in non-clinical participants who are high and low in delusional ideation (this will be subject to a separate review; Taylor, Hutton and Dudley, in preparation).

With regards the task variables, performance by people with and without non-affective psychosis will be considered on ‘easy’ versions of the beads task which will be defined as tasks using 85:15 ratio, and in a slightly broader criteria those studies using ratios of 90:10, and 80:20. Conversely, ‘hard’ versions of the beads tasks are defined as those that use the 60:40 ratio [4]. Additionally, the effect of salient or emotional variants of the tasks will also be considered [1]. Methodological factors, such as whether there are practice trials or multiple trials, will be addressed. Finally, whether an experimenter is present at the time of testing or whether the task is fully automated will be considered to test the hypothesis that raters might inadvertently cue an early response from people with psychosis, as in the ‘Clever Hans’ phenomenon; [17]. In summary, the moderators of effect size we intend to test are as follows:

• Whether the participants in the psychosis group also have delusions (delusions present versus absent)

• The stage of the psychosis (early psychosis versus chronic psychosis)

• Whether the participants were reporting current delusions or were remitted

• The difficulty of the JTC task (easy versus difficult task)

• The saliency of the JTC task (emotionally salient versus standard JTC task)

• The blinding of the researcher during JTC task (blind-rater or automated task versus rater aware of participant diagnosis/delusions)

• The matching of participants on demographics

• Whether the JTC task employed practice trials and/or multiple trials.

## Method

The review will aim to adhere to the Preferred Reporting Items for Systematic Reviews and Meta-Analyses (PRISMA) guidelines, [18] and the Assessment of Multiple Systematic Reviews (AMSTAR) guidelines [11] in order to ensure comprehensive and transparent reporting throughout.

### Inclusion and exclusion criteria

#### Population

Studies are required to recruit a sample of individuals either (i) with a diagnosis (*International Classification of Diseases, version 10* (ICD-10) or *Diagnostic and Statistical Manual of Mental Disorders, version 4* (DSM-IV)) of a schizophrenia spectrum condition (for example, schizophrenia, schizoaffective disorder, schizophreniform disorder, psychosis not otherwise specified (NOS)) or (ii) who experience delusions irrespective of condition (that is, trans-diagnostically). Including both types of sample enables us to explore the JTC bias at the more general level of psychosis versus non-psychosis and at the more specific level of delusions versus no delusions. This is crucial in establishing whether the JTC bias is specific to delusions *per se* or a correlate of psychotic illness more generally. The presence or absence of delusions may be based upon structured clinical interviews, such as the Positive And Negative Symptom Scale (PANSS); [19], or self-report measures, which may be more limited in terms of validity and reliability. The way in which delusions are assessed will be taken into account during the quality assessment (see below).

Studies will be excluded where ≥ 50% of the sample also have co-morbid diagnoses of bipolar disorder, learning disability, a primary diagnosis of substance-induced psychosis or psychosis secondary to a general medical condition or organic pathology.

For the purposes of the review we define first-episode or early psychosis as a first diagnosis of a schizophrenia-spectrum condition occurring within the last two years, following other research in this area [20].

The control groups may include both healthy controls and individuals with psychiatric conditions other than psychosis. Furthermore, in the cases of analyses comparing individuals with and without experiences of delusions, controls may include individuals diagnosed with psychotic disorders but who have not experienced delusions.

#### Design

A range of study designs will be suitable for inclusion. These include case-control studies whereby the cases may be defined either by the presence or absence of psychosis/delusions (where JTC bias would be the outcome), or by the presence or absence of a JTC bias (where psychosis or delusions would be the outcome). Cross-sectional correlational studies, whereby the relationship between JTC bias and continuous dimensions of delusional experience (for example, frequency, severity) is tested will be also be considered. Prospective designs where the relationship between psychosis or delusions and JTC bias is examined over time would also be eligible. Experimental designs whereby JTC bias is experimentally manipulated or trials of interventions aimed at improving JTC bias will be excluded. We will explore the impact of different study designs through sensitivity analyses.

#### Assessments of JTC bias

Studies employing either the beads task [21] or conceptual variants of this approach, for example, [22] will be included. Studies must report a measure of either (i) DTD, or (ii) a categorical index of JTC bias based on Garety and colleagues’ definition [5]. Studies that do not measure JTC but instead include a measure of ‘jumping to perceptions’ [7] or ‘jumping to attributions’ [8] will be excluded, as these tasks are not conceptual equivalents of the beads task.

#### Additional criteria

Only English language articles will be included. Unpublished data will also be included where identified.

### Search strategy

Electronic databases, including PsychInfo, PubMed, Medline and Web of Science, will be searched using the following keywords: (JTC or ‘jumping to conclusions’ or ‘jump to conclusions’ or ‘data gathering’ or beads or ‘probab* reason*’) AND (psycho* or schiz* or paranoi* or delusion* or persecut*). Searches will be undertaken using OVID search tools. Searches will go back as far as 1988, as this coincides with the first published study to use the beads task within psychosis (Huq *et al*. [21]). The electronic database searches will be undertaken independently by two authors (PJT, PH). Both authors are research psychologists specialising in clinical psychology with past experience of undertaking systematic reviews and meta-analyses.

References will be searched for all papers included in the review in order to identify any further relevant studies. In addition, the reviews of the JTC bias literature by Fine, and colleagues (2007), Dudley and Over [2], Garety and Freeman [3,23] and Freeman [9], will be examined to determine if any additional studies are included that were missed by our own literature searches. As a final step, all corresponding authors of included articles will be contacted and asked if they are aware of any further studies potentially meeting our criteria, including both recently published and unpublished studies.

### Study selection

In the initial phase, titles and abstracts will be screened for potentially eligible studies. In the second phase, full texts of the remaining articles will be read to determine if they meet the inclusion and exclusion criteria (all screening by PJT and PH). Where disagreement emerges regarding the eligibility of studies, a third author (RD) will arbitrate. Where conference abstracts that are identified through the search that possibly meet the inclusion criteria, the presenters will be contacted and further details sought regarding the study in order to ascertain the studies eligibility.

### Data extraction

Data will be independently extracted by two authors (PJT and PH) using a standard data collection form. Results will be compared and consistencies resolved with a third author (RD) acting as an arbitrator. Collected data will include sample characteristics (gender, age, ethnicity, clinical diagnosis, stage of illness, sample source and location), study design, JTC task (including bead ratio, number of trials, researcher blind or not, automated procedure or not), outcome data (for example, means, standard deviations, proportions of participants exhibiting JTC bias, correlations and regression weights where applicable).

### Methodological quality

We have adapted a tool for assessing the methodological quality of observational studies that has been successfully employed in prior research undertaken by the Agency for Healthcare Research and Quality (AHRQ) [24]. The main methodological quality criteria were retained but the underlying factors related to each study quality criterion were adapted in some instances for this specific context. Each study is assessed on a number of methodological quality criteria (for example, unbiased selection of groups, sample-size calculations, and so on) that are rated as being met, not met, partially met, or being unclear. A copy of this adapted measure is presented in Appendix 1.

Following the guidance of experts in the field of meta-analysis, we will avoid scale-based or aggregated study quality rating [25]. Quality assessments will be presented descriptively to guide the interpretation of findings, rather than used as a means to weight or adjust aggregated effect sizes. However, as noted, specific aspects of methodology will be tested as moderators of effect sizes. These will include blinding and the matching of participants on demographics.

### Assessing overall quality of evidence

The GRADE approach [26] was also adopted to provide an assessment of quality at the outcome level. Within the GRADE approach, observational studies are normally automatically marked down for quality. However, for the purposes of this review, as all included studies will be observational, a decision was made not to automatically mark down the quality of outcomes in this manner. All outcomes are therefore initially rated as high quality and then downgraded based on five main criteria (risk of bias, imprecision, indirectness, heterogeneity, publication bias).

The methodological quality of all studies and outcomes will be assessed independently by two authors (PJT and PH), with RD again acting as arbitrator. Both PJT and PH have completed the GRADE online learning modules (http://cebgrade.mcmaster.ca) and have experience of using this approach.

### Data synthesis and analysis

For studies comparing DTD between groups, Hedges’s *g* will be calculated, alongside the raw mean difference, as this index corrects for small sample bias [27]. Where means or standard deviations are not available to calculate effect size and associated standard error this will be estimated from other reported statistics (for example, *t, f*) or authors will be contacted for this information. Where JTC bias is treated as a binary variable, odds ratios (OR) will be calculated as an index of effect size.

Other research designs require additional consideration. Mean symptom severity data from studies comparing JTC and non-JTC clinical groups will not be compatible with studies comparing groups with and without psychosis/delusions. Where present, these data will be converted into OR, which will be used in the meta-analysis of binary outcomes. For studies employing correlational analyses, these will be converted to Hedges’s *g* (formula from Borenstein *et al*. [12]). Sensitivity analyses will be used to explore the impact of combining these converted effect sizes with other studies for the primary outcomes.

Where studies provide multiple comparisons between three or more different groups (for example, a group with psychosis, a psychiatric control group and non-clinical control group) a combined effect size will be estimated taking into account the correlation between effects (formula from Borenstein *et al*. [12]), and used in the meta-analyses. In addition, we will undertake a sensitivity analysis, re-calculating the meta-analyses to look specifically at comparisons with either psychiatric controls or non-clinical controls.

The presence of statistical heterogeneity will be assessed via the *Q*-test of statistical heterogeneity and quantified via the I^2^[28]. A random-effects model will be calculated within the meta-analyses as some degree of heterogeneity is expected across the studies. Nonetheless, where heterogeneity is moderate or less I^2^ < 40% [29], a sensitivity analysis will be undertaken examining the difference between fixed-effects and random-effects models.

### Subgroup analyses

As noted in the review aims, a number of moderators of effect size will be explored as part of the secondary outcomes of the review. These moderator effects will be examined through subgroup analyses. Significant differences between subgroups will be ascertained using the *Q*-test (Borenstein *et al*. [12]).

### Publication bias

Publication bias will explored via funnel plots for all outcomes with ten or more studies, following recommendations by Sterne, Egger and Moher [30]. The Trim and Fill method [29] will also be employed to explore the presence and ascertain the potential impact of publication bias. All analyses will be undertaken using Comprehensive Meta-Analysis [31] and STATA version 9 (Stata Corporation, College Park, TX, USA).

## Discussion

People with delusions are thought to have a data gathering bias in which they make decisions based on limited evidence. This ‘jumping to conclusions’ reasoning bias is thought to contribute to the development and maintenance of delusions. However, the robustness of this finding needs to be subject to systematic review and the extent of the effect considered with meta-analytic procedures. This proposed review will act as a definitive investigation of this well investigated process. In addition to quantifying the size of the JTC effect and its specificity to delusions versus psychosis in general, we also aim to determine whether a range of illness and task-related variables moderate the strength of the JTC effect. In light of this review, further development of theory and or clinical practice may well be warranted. In discussing the findings of the review, we will consider how they compare with the results of previous reviews in this area, their implications for future research and policy, the limitations and strengths of the review, and future research recommendations that can be drawn in light of the limitations of the available evidence.

## Appendix 1: quality assessment tool

General instructions: Grade each criterion as ‘Yes’, ‘No’, ‘Partially’, or ‘Can’t tell’. Factors to consider when making an assessment are listed under each criterion. Where appropriate (particularly when assigning a ‘No’, ‘Partially’, or ‘Can’t tell’ score), please provide a brief rationale for your decision (in parentheses) in the evidence table.

1. Unbiased selection of the cohort?

Factors that help reduce selection bias:

○ Inclusion/exclusion criteria:

○ Recruitment strategy

▪ Clearly described

▪ Criteria for inclusion in psychosis/delusions and comparison groups clearly outlined.

○ Recruitment strategy:

▪ Clearly described

▪ Relatively free from bias (selection bias might be introduced, for example, by recruitment via advertisement).

2. Selection minimizes baseline differences in prognostic factors?

Factors to consider:

○ Was selection of the comparison group appropriate?

○ Is the comparison group matched with the clinical group on key demographics (that is age and gender)?

3. Sample size calculated?

Factors to consider:

○ Did the authors report conducting a power analysis or describe some other basis for determining the adequacy of study group sizes for the primary outcome(s) of interest to us?

○ Where a power calculation is presented, do the final numbers obtained match up to this (for example, within 10% of required numbers)?

4. Adequate description of the cohort?

Consider whether the cohort is well-characterized in terms of baseline:

○ Age

○ Sex

○ Ethnicity

○ Diagnosis/clinical status

5. Validated method for ascertaining psychotic disorder or delusions?

Factors to consider:

○ Was the method used to ascertain exposure clearly described (details should be sufficient to permit replication in new studies)?

○ Was a valid and reliable measure used to ascertain exposure (subjective measures based on self-report tend to have lower reliability and validity than objective measures such as clinical interview)? Likewise, relying on medical notes is likely to introduce bias due to variation in how assessment is undertaken.

6. Validated method for ascertaining ‘jumping to conclusions’?

Factors to consider:

○ The beads task or a conceptually equivalent variant should be used

○ Were these measures implemented consistently across all study participants?

○ Were several trials and/or a practice run included in the procedure?

7. Outcome assessment blind to exposure?

Factors to consider:

○ Were the study investigators who assessed outcomes blind to whether participants had a psychotic disorder or delusions (this criterion will not apply in the case of Internet-based or automated designs where a researcher is not present)?

8. Adequate handling of missing data?

Factors to consider:

○ Are the details of missing data clearly reported, including how missing data was handled in the analyses? If not, is there any reason to believe missing data was present (for example, lower N in analysis than initially reported in the participants section).

○ Did missing data from any group exceed 20%?

○ If missing data was present and substantial, were steps taken to minimize bias (for example, sensitivity analysis or imputation).

9. Analysis controls for confounding?

Factors to consider for controlled studies:

○ If groups were not matched as baseline, did the analysis control for any baseline differences between groups?

○ Does the study identify and control for important confounding variables and effect modifiers (for example, IQ)?

10. Analytic methods appropriate?

Factors to consider:

○ Was the kind of analysis done appropriate for the kind of outcome data (categorical, continuous, and so on)?

○ Was the number of variables used in the analysis appropriate for the sample size (the statistical techniques used must be appropriate to the data and take into account issues such as controlling for small sample size, clustering, rare outcomes, multiple comparison, and number of covariates for a given sample size)?

## Abbreviations

JTC: Jumping to conclusions; DTD: Draws to decision; CBT: Cognitive Behavioural Therapy.

## Competing interests

The authors declare that they have no competing interests.

## Authors’ contributions

PJT: conception and design of the review methodology, writing of the protocol, critical revision and final approval of manuscript. PH: conception and design of the review methodology, critical revision and final approval of manuscript. RD: conception and design of the review methodology, writing of the protocol, critical revision and final approval of manuscript. All authors read and approved the final manuscript.

## Authors’ information

All three authors have had clinical and research experience working with individuals with psychosis. PH and PJT have worked together previously on systematic reviews regarding treatments for psychosis, whilst RD has produced several previous publications specifically in the field of the ‘jumping to conclusions’ bias in psychosis.
